# The Influence of an Acute Endurance Intervention on Breast Cancer Cell Growth—A Pilot Study

**DOI:** 10.3390/ijms26093976

**Published:** 2025-04-23

**Authors:** Nadira Gunasekara, Dorothea Clauss, Anika Voss, Konstantin Schurz, Katharina Fleck, Pablo Neu-Gil, Wilhelm Bloch

**Affiliations:** 1 Department of Molecular and Cellular Sports Medicine, Institute of Cardiology and Sports Medicine, German Sport University Cologne, 50933 Köln, Germanyw.bloch@dshs-koeln.de (W.B.); 2 German Cancer Research Center (DKFZ), 69120 Heidelberg, Germany; 3 Medical University Graz, 8010 Graz, Austria; 4 Medical University Innsbruck, 6020 Innsbruck, Austria

**Keywords:** exercise oncology, myokines, sports medicine

## Abstract

Exercise potentially inhibits tumor growth. It remains unclear which processes mediate these effects. Alterations of cytokine concentration in serum can influence cancer cell growth and may cause cell growth inhibition. This pilot study examines whether exercise-induced conditioning in serum can directly affect tumor cells. It focuses on serum collected before and after acute endurance exercise and its impact in vitro. Participants underwent a 1 h endurance training on a cycle ergometer. Samples were collected before, after, and two hours post-exercise. MDA-MB-231 cells were incubated with serum, and cell vitality and proliferation were assessed. Cytokine arrays identified relevant cytokine concentration changes. After identifying CXCL9 as a possible contributor to inhibitory effects, we inhibited the CXCR3 pathway and reassessed vitality. Exercise-conditioned serum significantly reduced cell vitality and proliferation post-intervention and after resting. Cytokine arrays revealed changes in multiple concentrations, and the inhibition of CXCL9 resulted in growth inhibitory effects. Our findings suggest that serum conditioned by an endurance intervention causes changes in cancer cell growth. Based on our observations, the alterations in serum cause growth-inhibitory effects, possibly mediated through the CXCR3 axis. This study provides preliminary evidence supporting the role of exercise in modulating the cancer cell growth directly by changes in serum.

## 1. Introduction

In women, breast cancer is the most common cancer type, with approximately two million new cases diagnosed each year [1]. Exercise is regarded as a component of cancer prevention as it contributes to lower incidences [2]. Exercise is an important component during cancer rehabilitation and is recommended as part of breast cancer treatment [3,4]. Apart from reducing disease- and treatment-related side effects in humans, numerous animal studies report a decrease in tumor weight and tumor growth, an increased immune response, and a reduced pathological score following exercise interventions [5,6,7]. An indirect effect on cancer cell growth through exercise via the immune system, mediated by cytotoxic T-cells, has been shown in animal studies [8]. In human studies, it is essential to identify the direct impact of exercise and the cytokines released on cancer cell growth. Additionally, the underlying mechanisms driven by myokines need to be investigated [9]. Regular physical activity promotes the mobilization and function of immune cells, such as cytotoxic T-cells and natural killer (NK) cells that infiltrate tumors and help fight cancer. Exercise also improves the body’s cytokine profile, enhancing the immune response in the tumor microenvironment (TME) while reducing the presence of immunosuppressive cells like myeloid-derived suppressor cells (MDSCs) [10,11]. The most commonly described factors contributing to changes in the TME are increased vascularization, enhanced immune surveillance, and metabolic changes in cancer-muscle cross-talk [12,13]. The communication between the tumor itself, cancer cells, and muscles is mediated by myokines, which are classified as a subcategory of cytokines that are released by contracting muscle tissue and, thus, released during exercise. Myokines are known to act as communicators between the muscle and other organs or within the muscle itself to promote hypertrophy. When released into the periphery, myokines have effects on multiple tissues. This applies to both healthy and cancerous tissues [14]. While these aspects may each be explored individually, there is likely an interaction among them [15]. The extent to which these mechanisms slow breast cancer growth remains unclear, and it is uncertain which type of exercise has the greatest impact. While there is evidence that exercise benefits the immune system and TME, further research is required to determine the optimal exercise approach [16].

The direct impact of metabolites and cytokines released during exercise on the TME may partly explain the observed effects of exercise on cancer. These molecules, such as interleukins and myokines, can modulate immune activity, enhance anti-tumor responses, and influence the TME by promoting immune cell infiltration and altering the inflammatory status. Moreover, a direct regulation of cancer cell growth by myokines should be taken into account. This interaction may be one of the mechanisms by which exercise indirectly supports tumor suppression in various cancer types. However, further research is needed to understand the extent of these effects [13].

The main components of the TME are the extracellular matrix, immune cells, stromal cells, and cells of the vascular system, with malignant and non-malignant cells surrounding the tumor and forming the TME through interactions with various surrounding cells, though its exact composition varies depending on the tumor type [17]. All of these components may be influenced by myokines [18]. The most well-characterized myokine is interleukin-6 (IL-6), which plays a central role in promoting muscle hypertrophy [14]. In the context of cancer, IL-6 has been recognized as directly affecting metastatic processes by altering the TME through multiple pathways that promote metastasis. Other direct effects of myokines on cancer cells include the induction of an epithelial–mesenchymal transition and the modulation of STAT3 signaling, thereby increasing proliferation. On the other hand, myokines can decrease cell migratory properties by increasing caspase-3/7 activity [16,19]. The increased expression of caspase-3/7 activity by altered myokine concentrations following resistance exercise has already been demonstrated in breast cancer cells that were treated with conditioned serum of breast cancer patients [19]. While the influence of muscle metabolites, such as lactate, has already been demonstrated, the influence of myokines via the immune or endocrine system is also likely, as myokines affect multiple tissues [15,20,21]. While myokines can have direct effects on cancer cells and the TME, as described above, indirect effects should also be taken into account [14]. Recently, Rundqvist et al. [8] demonstrated in a breast cancer animal model that muscles produce molecules, which caused an enhanced efficiency of CD8^+^ T-cells and resulted in a decrease in tumor growth. In a different study investigating a murine breast cancer model, mice that performed continuous endurance exercise exhibited lower tumor volume and decreased levels of IL-6 and vascular endothelial growth factor (VEGF) compared to a control group [22,23]. In cancer survivors, changes in the concentration of several myokines have been observed following acute high-intensity endurance interventions [24]. As there is a cross-talk between the muscle and the tumor, it is reasonable to assume that myokines play a role in TME regulation and, consequently, tumor growth [25]. In this context, the alteration of cancer cell extracellular matrix by myokine-regulated expression of matrix metalloprotease (MMP), which can enhance migratory properties, should be considered [26]. Rundqvist et al. demonstrated that incubation of prostate cancer cells with post-exercise pooled serum inhibited cancer cell growth in vitro*,* a finding confirmed in a murine model [27]. Beyond myokines and cytokines, metabolic changes from exercise-induced lactate fluctuations should also be considered when investigating the effects of conditioned serum on cancer cells [28]. Higher than normal lactate concentrations within the TME may favor the immune system escape while also providing an energy source for cancer cells [29]. In contrast to these findings, higher lactate concentrations in the tumor environment also have the potential to support CD8^+^ cells in developing stem-cell-like characteristics and thereby increasing anti-tumor immunity [30].

As breast cancer is the most common cancer among women, and positive effects of exercise on cancer- and treatment-related side effects have been shown in humans, it is reasonable to explore whether exercise-conditioned serum and associated cytokines directly influence the growth of breast cancer cells [31]. Therefore, the purpose of this pilot study is to identify whether conditioned serum has an effect on breast cancer cell growth in vitro. Secondly, we tested whether changes in protein or cytokine concentrations within the serum can be detected. We further explored whether the concentration changes we observed could be directly linked to growth inhibition. This pilot study serves as a basis for future research to build upon.

## 2. Results

### 2.1. Cell Proliferation

We compared cell proliferation in cells that were treated with pre-exercise (T0_intervention_), post-exercise (T1_intervention_), and rest serum (T2_intervention_). An immunohistochemistry with Ki-67 was performed. The ratio of Ki-67-negative to Ki-67-positive cells as well as the relative change of Ki-67-negative vs. Ki-67-positive cells from T0_intervention_ to T1_intervention_, T1_intervention_ to T2_intervention_, and T0_intervention_ to T2_intervention_ was calculated. For this purpose, two independent researchers counted 100 cells per participant at each time point. The relation of negative cells to cells counted in total was assessed for each participant, and means were calculated for each condition. We found statistically significant differences between the proliferation rates of each time point (*Chi^2^* (2) = 20.182, *p* < 0.001, *n* = 11). We found that the proliferation rates decreased from T0 to T1 (*p* = 0.032) as well as from T0 to T2 (*p* < 0.001), but not from T1 to T2 (*p* = 0.165). The effect sizes of T0–T1 (*r* = 0.315) and T0–T2 (*r* = 0.417) showed medium efficiency according to Cohen’s classification, while T1–T2 (*r* = 0.273) showed low efficiency [32].

### 2.2. Analysis of the Cytotoxic Effects

**Figure 1:**
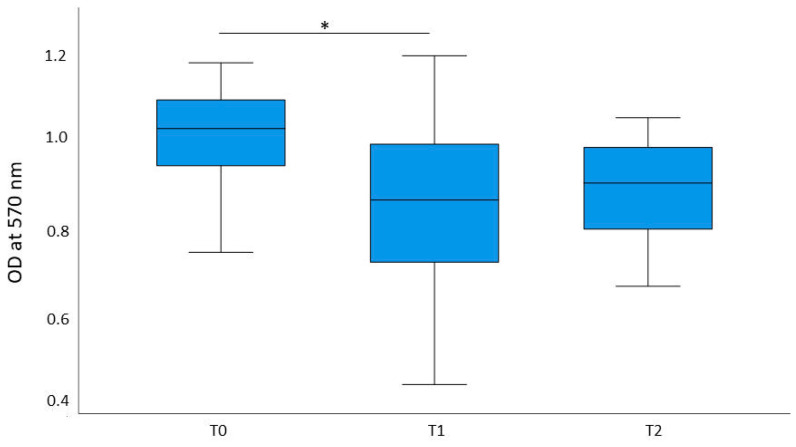
Results of the MTT assay. MDA-MB-231 cells were treated with individual sera of all 11 participants and an MTT assay was conducted to assess cell viability. The blood samples were drawn before the endurance intervention (T0), directly after the endurance intervention (T1), and after 2 h of rest (T2). The optical density (OD) was measured at 570 nm. Median values of the optical density measured during MTT assay for T0, T1, and T2 with serum taken on the day of the intervention are displayed. The mean values of each time point were tested for statistical differences. Statistically significant differences of mean values following an rmANOVA are marked in the figure (* = *p* < 0.05). Cell viability was significantly decreased in cells treated with post-exercise conditioned serum (T1) compared to pre-exercise (T0) and rest serum conditions (T2). Therefore, serum conditioned by an acute moderate intensity endurance exercise decreased cell viability in MDA-MB-231 cells.

Cell viability was determined via the optical density (OD) measured following an MTT assay. Cell viability was highest in the pre-exercise condition (T0) (*M* = 1.05, *SD* = 0.194), followed by the rest condition (T2) two hours after the endurance exercise (M = 0.924*, SD* = 0.164). The lowest cell viability was measured immediately after the intervention at T1_intervention_ (*M* = 0.887, *SD* = 0.218), as shown in Figure 1. Significant differences in cell viability were found between the time points T0_intervention_ and T1_intervention_, as determined by an rmANOVA (*p* = 0.046), indicating that cell viability is significantly reduced in cells treated with conditioned serum from blood which was drawn directly after the intervention.

### 2.3. Cytokine Array

After processing the protein arrays according to the manufacturer’s instructions with pooled serum for each condition, a 2D densitometry analysis was conducted on cytokine array membranes using ImageJ. Signal intensities of individual cytokine spots were quantified from scanned images. Values were normalized to internal controls for comparative analysis. The following cytokines were selected for densitometry analysis: Leptin, PDGF-BB, CCL5, MCP1, IL-15, Angiogenin, BDNF, TNF- α, CXCL9, IL-3, CCL 15, IGFBP-1, NAP-2, EGF, IGFBP-2, Eotaxin-1, ICAM-1, TIMP-1, EGFR, TIMP-2, CXCL5, IGFBP-6, CCL4, TRAILR3, Adiponectin, MIP-3-beta, MSP, uPAR, ANGPT2, OPG CXCL1, gp130, HCC-4, and IL-6 R. Full cytokine names can be viewed in the list of abbreviations.

Densitometry was analyzed for intervention and control conditions to determine if differences were due to the intervention or factors like circadian rhythm. A Friedman Test assessed whether changes between T0–T1, T1–T2, and T0–T2 were exercise-related or influenced by other factors. We compared absolute densitometry values of each condition and time point (*Chi^2^* (5) = 28.353, *p* < 0.001, *n* = 34) and a post hoc analysis, which showed significant differences between values for all cytokines combined at T1_rest_ and T1_intervention_ (*p* < 0.001) and T2_rest_ and T1_intervention_ (*p* < 0.001) but not between T0_rest_ and T0_intervention_ (*p* = 0.846) and T2_rest_ and T2_intervention_ (*p* = 0.92). Changes in concentrations are presented in Table A2.

**Figure 2:**
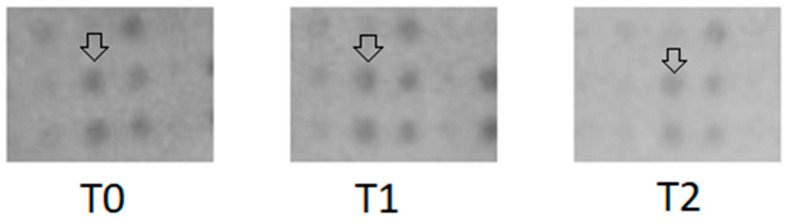
Cytokine array results for CXCL9. Arrays were incubated with pooled serum from 11 participants at three time points: pre-exercise (T0), post-exercise (T1), and after rest (T2). A control was included in the experiment but is not shown in this figure. The excerpt presented highlights the CXCL9 spot, indicated by the arrows. A gradual decrease in CXCL9 concentration across time points is visible and was quantified using densitometric analysis in ImageJ (V. 1.51j8). All arrays were processed and imaged under identical conditions.

Cytokine concentrations were similar between intervention and control conditions at the first serum collection time point. The exercise intervention altered cytokine levels compared to the control condition, where participants rested for 1 h instead of cycling. Figure 2 presents an excerpt of the array with CXCL9 marked in the pre-exercise condition, post-exercise condition, and after resting.

Repeating the analysis of change rates confirmed significant differences between T0_rest_-T1_rest_ and T0_intervention_-T1_intervention_, while other changes were not statistically significant. Thus, densitometry changes are likely due to the exercise intervention and independent of other factors like the circadian rhythm or nutritional status.

### 2.4. Blocking CXCR3 with AMG 487

Previous experiments showed decreased concentrations of multiple cytokines when comparing pre- vs. post-exercise serum. MTT assays and immunohistochemistry revealed significant differences in cell proliferation and vitality from T0_intervention_ to T1_intervention_ and between intervention and control conditions. Given CXCL9′s role in the TME, we investigated whether reduced cell activity was linked to CXCL9 concentration [33]. To test this, we used AMG 487, a CXCR3 antagonist, to block the CXCL9 pathway and repeated cytotoxicity and proliferation assays with pre-exercise (T0) serum and T0 serum plus an inhibitor (T0 + IH).

#### 2.4.1. Analysis of the Cytotoxic Effects

**Figure 3:**
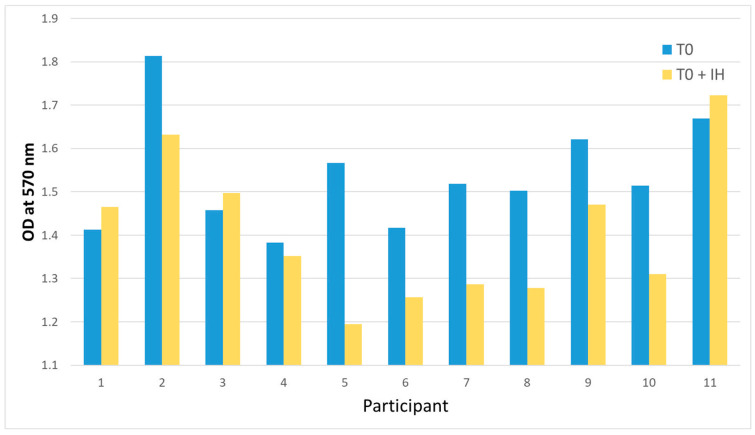
Results of the MTT assay after the inhibitor experiment. An MTT assay was performed on MDA-MB-231 cells, which were treated with each participant’s pre-exercise (T0) serum and each participant T0 serum plus the CXCR3 pathway inhibitor AMG 497 (T0 + IH). Optical density was measured at 570 nm. Except for the serum of two participants, cell viability decreased in the T0 + IH condition, which was confirmed by a two-sided paired *t*-test.

The inhibition of the CXCR3 axis resulted in reduced cell viability. OD was determined at 570 nm for cells incubated in the T0 condition (T0) and the T0 condition with an inhibitor (IH). Cell viability at T0 (*M* = 1.53, *SD* = 0.07) was higher than T0 + IH (*M* = 1.406, *SD* = 0.14). The analysis yielded a t-value of *t* (10) = 3.064 and a *p*-value of *p* = 0.012. Figure 3 shows that the cell viability was significantly reduced in cells that were treated with the T0 serum and the inhibitor in individual participants. Two participants had opposing effects, which did not affect the overall results. This might be due to a measurement error or individual responses of said participants.

#### 2.4.2. Proliferation

**Figure 4:**
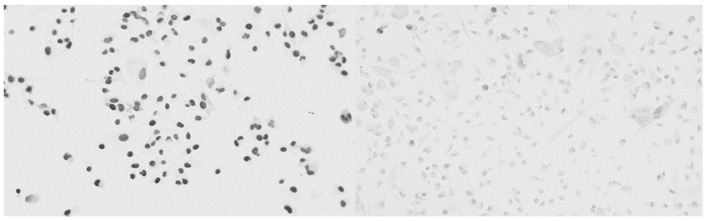
Immunohistochemistry results before and after inhibition of CXCR3. On the left, MDA-MB-231 cells were treated with the conditioned pre-exercise serum (T0) and Ki-67. The darker appearance of the cells indicates the presence of Ki-67, an indicator of proliferative cells. On the right, cells were treated with the conditioned serum of the pre-exercise condition (T0), Ki-67, and CXCR3 inhibitor AMG 486. These samples stem from one participant, but experiments were performed and evaluated for all conditions and all 11 participants. It became evident that cells treated with the CXCR3 inhibitor express less Ki-67 and therefore lack proliferative properties. This was confirmed by a two-sided paired *t*-test. Images were taken at 20× magnification.

**Figure 5:**
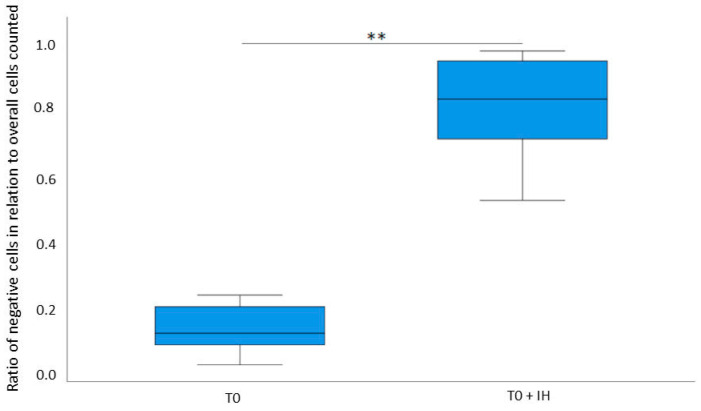
Statistical analysis of immunohistochemistry results before and after inhibition of CXCR3. This boxplot shows the results of the immunohistochemistry, where MDA-MB-231 cells were treated with pre-intervention (T0) conditioned serum and T0 conditioned serum with CXCR3 partway inhibitor AMG 487 (T0 + IH). All participants were included. Images of the slides were analyzed with ImageJ (V. 1.51j8), and cells were counted by two independent observers. The median and distribution of the ratio of non-proliferative to overall counted cells in the conditions T0 and T0 + IH are shown. Two outliers at T0 were excluded for graphical reasons but were included in the statistical analysis. The mean values of the two groups were compared by a paired two-sided test. The T0 + IH condition shows a significantly higher count of non-proliferative cells. Therefore, inhibition of CXCR3 inhibited cell proliferation. Statistically significant differences are marked (** = *p* < 0.01).

Inhibition of the CXCR3 pathway reduced cell proliferation. MDA-MB-231 cells were treated with conditioned serum of T0 and T0 serum with the inhibition of the CXCR3 pathway by AMG 487 (T0 + IH). A Ki-67 immunohistochemistry was performed for both conditions. Consequently, we compared the conditions T0 and T0 + IH and found that the cell proliferation rate was reduced in the cells that were treated with the T0 + IH serum (*t* (10) = −11.734, *p* < 0.01). Figure 4 shows an example of microscopic imagery, while Figure 5 displays the statistical results. When inhibiting the CXCR3 pathway in pre-exercise serum, cell proliferation was significantly reduced. This was similarly observed in the post-exercise condition.

In summary, treating MDA-MB-231 cells with serum from healthy, minimally active women after acute endurance exercise reduced cell vitality and proliferation. Follow-up experiments using a baseline serum with a CXCL9 pathway inhibitor also reduced cell viability and growth, highlighting the relevance of CXCL9 in the context of exercise effects on cancer cells.

## 3. Discussion

We could confirm that breast cancer cell growth decreased after being treated with the conditioned serum of healthy women drawn immediately after a 1 h moderate intensity endurance exercise session. This demonstrates the feasibility of the growth inhibitory effects of conditioned serum after exercise, which was the primary aim of this pilot study. Additionally, we examined the concentration and changes in multiple cytokines in the serum at different time points. We observed concentration changes in multiple cytokines across all time points, accompanied by decreased proliferation and vitality. The densitometry results indicated that these concentrations also fluctuate in the control condition, albeit to a lesser extent.

Acute and chronic endurance exercise interventions are recognized as important components in influencing tumor growth. In cancer survivors of multiple entities, it was demonstrated that concentration changes in myokines occurred acutely after a high-intensity session but not chronically [24]. Exercise is generally associated with beneficial outcomes in various cancer types, and emerging guidelines suggest that exercise can be effectively integrated into cancer treatment [34]. Breast cancer patients undergoing treatment are advised to engage in 150 min of moderate-intensity endurance exercise weekly, based on its benefits for HRQoL. Combined data from multiple studies suggest that endurance exercise may also affect survival rates [35]. Despite these recommendations, it remains uncertain which type of exercise or dosage is most beneficial in directly affecting the tumor, as the studies investigating this topic are heterogeneous [36,37,38]. The promising effects of resistance exercise on myokine concentration and, consequently, growth inhibition in triple-negative breast cancer cells have already been demonstrated [19]. In our pilot study, we showed that acute endurance intervention at moderate intensity had an inhibitory effect on triple-negative breast cancer cell growth in vitro [27]. Known effects of endurance exercise that can reduce tumor growth include a reduction in C-reactive protein (CRP) levels and the release of inflammatory markers such as IL-6, sex hormones, insulin response, and vascularization [21,39,40]. Besides these factors, immune cells play a vital role not only in peripheral tissues but also within the TME, where they can either promote or inhibit tumor progression [41].

Although these effects impact the tumor, they may not be directly responsible for the reduction in cancer cell growth observed with exercise. In a study comparing the effects of an acute exercise intervention to a six-month exercise intervention in breast cancer patients, reduced cancer cell growth in vitro was only present in cells treated with serum after the acute exercise intervention. Although cytokine changes in both sera were detected, cell viability in MDA-MB-231 cells remained the same when treated with the chronic condition serum. While the effects of chronic exercise favor a positive outcome and generally lower inflammatory environments, the authors concluded that the accumulated effects of acute exercise can directly inhibit breast cancer cell growth [42]. Our results support these findings. The release of myokines from the muscle into the periphery during and after exercise and their function in the TME may be one factor contributing to cancer cell growth inhibition. While research on myokines is still limited, they likely influence oncogenic pathways as ligands or activate suppressor pathways directly [40]. The relevance of myokines was additionally highlighted by a pilot study, which found that myokine concentrations are altered by exercise in breast cancer patients [43]. Supporting these findings, this pilot study found that serum from sedentary women, conditioned by acute endurance exercise, inhibited breast cancer cell growth immediately after the intervention but not after two hours of rest. Cytokines, which could mediate this exercise-dependent effect on triple-negative breast cancer cell growth, were identified, and we focused on the CXCR3 axis in further experiments. Additionally, we took the lactate concentration within the serum into account. Higher lactate concentrations within the TME are associated with tumor growth and metastasis formation, when the lactate is produced by cancer cells as an energy source [44]. When higher lactate concentrations from external sources surround cancer cells, cell proliferation is inhibited [45]. We saw inhibitory effects in cells that were treated with higher lactate concentrations after the intervention, but those effects were irrelevant for the inhibition of the CXCR3 pathway, as cells were treated with pre-exercise serum and added inhibitor. Therefore, our observations are likely independent of the serum lactate concentration.

We found that the concentration of CXCL9 was lower after exercise. Therefore, we chose to inhibit the CXCL9 pathway via its receptor CXCR3, as this cytokine has been described as influencing tumor cell growth [33]. This approach was previously demonstrated by Liu et al. (2011) [46]. In this condition, we also observed growth inhibitory effects. CXCR3 is a cell membrane receptor that can be activated by CXCL9, C-X-C motif chemokine 10 (CXCL10), and C-X-C motif chemokine 11 (CXCL11). The two major mechanisms in which CXCR3 and its ligands are involved are angiogenesis and the recruitment of different immune cells at inflammatory sites [47]. There are two common isoforms of the CXCR3 receptor, CXCR3 A and CXCR3 B. Overall, CXCR3 is associated with cancer progression by altering the TME, but also with anti-tumor effects and growth reduction in vivo [48,49]. In a recent study, the CXCR3 A isoform was associated with modulation of cancer stem-cell-like properties, leading to cancer progression and treatment resistance. Isoform B is also associated with inhibiting effects, while this isoform can also have tumor-promoting effects, but only when it is overexpressed [48]. Breast cancer cells, including MDA-MB-231 cells, typically express CXCR3 A receptors [50].

The interaction of ligands with CXCR3 can be described as ambiguous. The ligands CXCL9, CXCL10, and CXCL11 all bind to CXCR3 and are primarily secreted by immune cells such as leukocytes and macrophages, as well as by dendritic cells, fibroblasts, and tumor cells. These cytokines are normally expressed at low levels but are upregulated in response to inflammation [33]. CXCL9, CXCL10, and CXCL11 interact with CXCR3 on tumor cells, immune cells, and vascular endothelial cells, influencing tumor growth, immune cell activity, and blood vessel formation. Tumor cells can secrete these cytokines and bind them to their receptors in an autocrine manner, promoting tumor growth and metastasis. Additionally, this signaling axis regulates the immune response, enhancing the activity of immune cells and inhibiting angiogenesis. Another important component of the acute effects of exercise is the activation of the p53 protein, which causes apoptosis [51]. The p53 and CXCR3 pathways may intersect, as p53 activation can regulate immune responses by modulating cytokine signaling, potentially enhancing CXCR3-driven T-cell recruitment. Furthermore, CXCR3 expression correlates with better cancer prognosis in some cases, suggesting that exercise-induced p53 activation and immune modulation could synergize to suppress tumor growth [52,53].

The effects of the CXCL9/CXCL10/CXCL11-CXCR3 axis can vary, even within the same organ, depending on the distribution of CXCR3 subtypes in tumor cells. For instance, in breast cancer patients, elevated serum levels of CXCL9 and CXCL10 are observed compared to healthy controls, and high levels of CXCL9 transcripts have been linked to better outcomes. While it is apparent that the CXCR3/CXCL9 axis is important in cancer, it may depend on the target cell if the interaction has a progressive or inhibitory effect on the cancer cell [54]. In a murine breast cancer model, different ligands of the CXCR3 receptor were elevated in varying breast cancer types after exercise. In this case, multiple cell types expressed CXCR3, CXCL9, and other ligands, but there was no predominant cell type [55]. When interacting with CXCR3, CXCL9 promotes tumor growth and metastasis in certain contexts, but it also recruits immune cells like cytotoxic T-cells to the tumor, supporting anti-tumor immunity. CXCL9, therefore, can either promote or inhibit tumor progression by influencing the tumor microenvironment. This complex behavior currently makes CXCL9 a controversial target in cancer therapy [56]. It is suggested that targeting this pathway might prevent? immune system evasion by enhancing the recruitment of immune cells into the TME via the CXCR3 axis [49]. In another study, CXCL9 induced cancer cell growth in an in vitro model of human and murine glioma. At the same time, the inhibition of CXCR3 had an anti-tumor effect in this model, similar to our observations [46]. Coherent with our results, CXCL9 was reduced in mice that exercised compared to mice that did not [57].

As cancer cells can produce CXCR3 ligands themselves, it appears plausible that cancer cells utilize this loop to gain cancer stem-cell-like properties, improve proliferation, promote metastasis, and immune system evasion [54]. Along with our pilot study, studies showed that an inhibition of this loop has an inhibitory effect on cancer cell growth, and the CXCR3 axis is proposed to be a target for treatment [56]. This pilot study supports the notion that inhibition of the CXCR3 axis has an inhibitory effect on breast cancer cell growth. Additionally, we showed that an acute endurance exercise decreases CXCL9 concentration in serum. Consequently, exercise may have a direct effect on breast cancer cell growth via the CXCR3 axis, although this was not explicitly demonstrated in our study. However, we could show that serum conditioned by an acute endurance intervention has an inhibitory effect on triple-negative breast cancer cell growth. This indicates that changes in cytokine concentration within the periphery are causing these effects. We suggest further exploration of the CXCR3 axis, its ligands, and exercise in multiple cancer models, as there is a strong link between this pathway and the direct effect of exercise on cancer cells.

### Limitations

As our study served as a pilot study, a limitation is the small number of participants. It would be beneficial to apply our findings to a larger population to validate them or to repeat our experiments with serum of cancer patients before and after exercise. Despite choosing two independent researchers and following straightforward guidelines, the interpretation of microscopy images may be subjective. We made an effort to avoid this by calculating the interrater reliability before further analysis. As we explored the overall changes in cytokine concentration via cytokine arrays, these analyses were not as exact as other methods, but as we were trying to investigate a large number of cytokines, it was reasonable for a pilot study. In future studies with more defined research questions, other types of analyses are preferable, and other cytokines should be investigated. Additionally, it is necessary to repeat the experiments on a different cell line to validate the effect of exercise. In our experiments, while the serum used to treat the cells was paired for each individual, the cells themselves were not. This limits our ability to track individual cellular responses. This lack of pairing might result in a loss of contextual information and increased variability. Therefore, statistical power is reduced, requiring larger sample sizes in future studies to verify statistical significances observed in this pilot study. Additionally, since the analysis captures only a fraction of cellular states, identifying dynamic changes remains challenging despite the controlled serum conditions.

## 4. Materials and Methods

### 4.1. Participants and Study Design

This study was approved by the ethics committee of the German Sport University Cologne and follows the Declaration of Helsinki on 12 April 2023 under the agreement number 041/2023. All participants were informed about the study and provided written informed consent prior to baseline testing.

The observations of Rundqvist et al. [27] were used to calculate the number of participants required. As we know of no comparable studies, a power of 0.8 and an α-error of 0.05 were assumed for a group size of 12 test subjects, which was initially intended. Compared to Rundqvist et al. [27], two additional subjects were planned to be recruited. This resulted in an effect size of 0.89 and was calculated with G-Power for 12 participants.

A crossover design was chosen, and participants were randomized into one of two groups, A and B, after inclusion. One person withdrew consent, and the data were excluded. This resulted in *N* = 6 in Group A and *N* = 5 in Group B. The inclusion criteria were a physical activity level below 210 min of moderate intensity per week and no history of cancer disease, as well as no other currently treated disease or medication intake. Exclusion criteria were ages below 30 and over 60, presence of any chronic disease, and acute injuries. Participant characteristics are displayed in Table A1.

Upon inclusion, participants completed the Physical Activity Readiness Questionnaire (PAR-Q) and International Physical Activity Questionnaire (IPAQ) in German to validate inclusion criteria [58,59]. Consequently, they performed Cardio Pulmonary Exercise Testing (CPET), utilizing a ramp protocol on a cycle ergometer to determine peak aerobic capacity (VO_2peak_). The initial load was 20 watts, which increased by 10 watts per minute. The assessment was discontinued when a rating of perceived exertion (RPE) of 20 was reached or upon voluntary termination. All measurements were completed within a four-week time window. The objectives of this study were to identify, if conditioned serum by an acute endurance intervention has a direct effect on breast cancer cell growth in vitro. Furthermore, we aimed to identify relevant cytokines in serum and verify these findings with an inhibitory experiment.

### 4.2. Intervention

The intervention consisted of cycling on an ergometer for a total of 60 min, 20 of which were completed at 50% VO_2peak_ and the remaining 40 min at 60% VO_2peak_ [27]. Venous blood collection was performed directly before (T0_intervention_), after (T1_intervention_), and two hours after (T2_intervention_) the intervention. Participants were advised to remain seated or lying down during the resting period. At the second study appointment, precisely one week after (Group A) or before (Group B), the intervention blood draws were completed at the same time points (T0_rest_, T1_rest_, T2_rest_). Instead of cycling, participants rested for 60 min. Participants were asked to avoid alcohol and strenuous exercise 24 h before testing and caffeine 2 h before and during testing.

Upon collection, serum was isolated from the blood samples and stored at −80 °C until further use.

The MDA-MB-231 is a triple-negative, metastatic breast cancer cell line, which was kindly provided by the American Type Culture Collection (ATCC^®^, Manassas, VA, USA). The cells were cultured in medium consisting of 89% Dulbecco’s Modified Eagle Medium (high glucose, Gibco™ (Thermo Fisher Scientific, Grand Island, NY, USA)), 10% fetal bovine serum (FBS, Gibco™), and 1% Penicillin-Streptomycin (pen/strep, Gibco™). Cells were incubated at 37 °C and 5% CO_2_.

### 4.3. Analysis of the Cytotoxic Effects

We incubated MDA-MB-231 cells with medium containing the serum of individual participants from time points T0_intervention_, T1_intervention_ and T2_intervention_ to test for cytotoxic effects of the endurance intervention.

The MTT assays were performed in 96-well plates (Biofil^®^, Guangzhou, China) with an area of 0.32 cm^2^ per well. For each well, 20,000 cells were seeded in 100 μL medium of each condition from every participant. Each condition was performed twice per participant. After the cells were treated in the incubator for 48 h at 37 °C, 5% CO_2_, 10 μL of MTT reagent (10%, R&D Systems^®^, Minneapolis, MN, USA) was added to each well and incubated for 2 h 30 min in the incubator at 37 °C and 5% CO_2_. The medium was then removed, and 100 μL of dimethyl sulfoxide (DMSO) (Thermo Scientific™, Villebon-sur-Yvette, France) was added. After 2 h in darkness at 37 °C, the OD was measured at 570 nm (OD570) and a reference wavelength of 650 nm (OD650) using a multiscan photometer (Thermo Scientific™ Microtiter Plate Photometer Multiskan™ FC). We used a negative control.

### 4.4. Immunohistochemistry

Immunostaining with the anti-Ki67 antibody (Novus Biologicals^®^, Centennial, CO, USA) was performed in 24-well plates (Falcon^®^ (Becton, Dickinson and Company), Franklin Lakes, NJ, USA) with 1.09 cm^2^ per well. Manufacturer instructions were followed. After coating each well with Aclar platelets, 20,000 MDA-MB-231 cells were seeded in 1 mL medium per well. This resulted in three conditions, T_0_, T_1_, and T_2_ for each participant. The cells were treated with a medium containing conditioned serum for 48 h. The media consisted of 89% Dulbecco’s Modified Eagle Medium (high glucose, Gibco™) 10% serum (T0_intervention_, T1_intervention_, T2_intervention_), and 1% pen/strep (Gibco™). The medium was then removed, and the cells were washed twice with phosphate-buffered saline (PBS) and fixed with 4% paraformaldehyde at 37 °C for 15 min.

They were washed three times with PBS and stored for 7 days at 4 °C with 1 mL PBS per well. Each well was washed with 0.05 M TRIS-buffered saline (TBS), then incubated for 20 min with 3% hydrogen peroxide in methanol and washed again with 0.05 M TBS. After 10 min with 0.5 M ammonium chloride and 0.25% Triton X-100 (Sigma-Aldrich, St. Louis, MO, USA) in 0.05 M TBS and washing with 0.05 M TBS, 5% bovine albumin (BSA) in 0.05 M TBS was added for 60 min. The cells were incubated overnight with the anti-Ki-67-Rb antibody (NB500-170,j 1:250 in 1% BSA; Novus Biologicals^®^) at 4 °C and washed with 0.05 M TBS. A Goat anti-rabbit antibody (rabbit polyclonal to Ki-67, 1:400 dilution in 0.05 M TBS, Novus Biologicals^®^) was added to the cells for 60 min. After multiple washes with 0.05 M TBS, the cells were incubated for 60 min with the Horseradish peroxidase complex (1:400 dilution in 0.05 M TBS, Sigma-Aldrich) and washed again with 0.05 M TBS. The 3,3′Diaminobenzidine (DAB) solution mixture (150 μL DAB, 150 μL ammonium chloride, 300 μL nickel sulfate, 300 μL glucose, 5 μL glucose oxidase in 15 mL 0.1 M Pb, pH 7.4) was then added to the cells for 20 min. The reaction was stopped by removing the DAB solution and adding 0.05 M TBS. Finally, methyl green staining was performed. For this purpose, 200 μL of methyl green zinc chloride (1%, Sigma-Aldrich) was added to each well for 8 min and then washed with 0.05 M TBS and 96% ethanol. The water was removed using a descending series of alcohols (96%, 100% ethanol, and 100% xylene), and the glass plates with the cells were then transferred to slides.

Slides were analyzed under a light microscope. One researcher photographed them, while two others independently counted 100 cells per condition per participant. A fourth researcher evaluated interrater reliability by assessing the ratio of inactive to total cells for each condition and time point.

### 4.5. Cytokine Array

Cytokines in serum samples were screened using the RayBio^®^ Human Cytokine Array C1000 Series (RayBiotech, Inc., Peachtree Corners, GA, USA.). Undiluted pooled samples for T0_intervention_, T1_intervention_, T2_intervention,_ T0_restl_, T1_rest,_ and T2_rest_ were processed following the manufacturer’s instructions with minimal incubation times. Chemiluminescence was detected using the ChemiDoc^TM^ MP Imaging System (Bio-Rad Laboratories, GmbH, Feldkirchen, Germany). Cytokines with visually distinct concentrations across time points were further analyzed via 2-D densitometry in ImageJ.

### 4.6. Lactate Concentration

Lactate concentration was measured in 20 μL of serum per participant per time point using Eppendorf vessels and analyzed with the EKF Biosen C-line.

### 4.7. Blocking CXCL9

The previous experiments revealed a decrease of Chemokine (C-X-C motif) ligand 9 (CXCL9) in serum as portrayed by a protein array, along with a reduction in cell proliferation as determined by immunohistochemistry with Ki-67. Cell growth and vitality, as shown in the MTT assay and immunohistochemistry, differed significantly from the time points T0 to T1, and these differences were also significant in the intervention condition vs. the control condition.

As CXCL9 is associated with changes in the tumor growth, we further investigated if the reduction in cell activity is caused by changes in CXCL9 concentration in serum [33]. As we observed a reduction in CXCL9, we used AMG 487 (Biomol, Hamburg, Germany), a CXC chemokine receptor 3 (CXCR3) antagonist, to block the CXCR9 pathway. We replicated the MTT assay and immunohistochemistry with serum from the time points T0 and T0 + IH. We expected to observe similar results to previous experiments.

Tumor cells were seeded as previously described for the MTT assay and immunohistochemistry, and consequently treated with medium containing T0 serum or medium with serum from T0 containing 1 μmol/L AMG 487 for 48 h at 37 °C and 5% CO_2_ to compare the results to our previous ones [60].

AMG 487 was prepared as a 0.2 mM stock with DMSO according to the manufacturer’s instructions.

The MTT assay and immunohistochemistry with Ki-67 were performed as previously described using controls with FBS and DMSO.

### 4.8. Statistical Analysis

*Statistical analyses were performed using IBM SPSS Statistics (Version 29).*

For the cell proliferation assay, we first calculated the interrater correlation coefficient. We looked at the ratio of Ki-67-negative to Ki-67-positive cells and the changes from T0_intervention_ to T1_intervention_, T1_intervention_ to T2_intervention_, and T0_intervention_ to T2_intervention_. Two independent researchers counted 100 cells per participant at each time point, and the proportion of negative cells was used to calculate the intraclass correlation coefficient (ICC) with a two-way mixed model, assuming systematic error [61]. The results are shown in Table 1.

We tested the data for normality using a Shapiro–Wilk test and found they were not normally distributed. Therefore, we used a Friedman test to compare time points and performed a post hoc analysis with Bonferroni correction (significance level *α* = 0.05). We also calculated effect sizes. For the cytotoxicity analysis, normality was tested with a Shapiro–Wilk test, and sphericity with Mauchly’s test followed. Since the data from the cytokine array densitometry analysis were not normally distributed, we used a Friedman test to test if differences between T0–T1, T1–T2, and T0–T2 were due to exercise or other factors. We compared absolute densitometry values and performed a post hoc test with Bonferroni correction. For the cell proliferation essay, after blocking with AMG 487, we followed a similar approach, calculating ICCs. Both ICCs are good or very good (*T0* = 0.819, *T0* + IH = 0.652) [61]. We tested the data of the inhibitor experiments for normality with a Shapiro–Wilk test and compared the two conditions with a two-sided paired *t*-test. A similar *t*-test was also used to compare the conditions in the cytotoxicity analysis.

## 5. Conclusions

There are multiple known factors, like the immune and capillary systems, as well as cytokines and metabolites, which have an influence on tumor growth. In our pilot study, we show that there are variable components in serum conditioned by an acute endurance intervention. These factors in conditioned serum inhibited triple-negative breast cancer cell growth in vitro. Moreover, we identified a cytokine, CXCL9, which is likely regulated by exercise and may have a direct effect on cancer cell growth. These results align with the current literature on this topic. While chronic exercise interventions benefit overall health, the cumulative effects of individual exercise sessions may have the most significant impact on reducing tumor growth [40]. Our results support the theory that a single endurance intervention has an inhibitory effect on triple-negative breast cancer cell growth in vitro. Here, more highly standardized and homogenous studies investigating chronic vs. acute effects of different exercise modalities and different cancer cell lines are required to gain perspective. We could also support the finding that the CXCR3 axis has an inhibitory effect on tumor cell growth. Some studies also demonstrate the involvement of this axis in the TME, while other findings are contrasting [33]. More studies are required to understand this axis and the specific influence of exercise on it, as well as its subsequent impact on cancer. More factors should be analyzed individually and in combination, especially in cancer patient populations.

## Figures and Tables

**Table 1:** ICC for observation of immunohistochemistry. This table presents the results of the intraclass correlation analysis (ICC), which was conducted to assess the agreement between researchers in their observations of immunohistochemistry images.

Time Point	ICC ^1^	95% Confidence Intervals (Lower/Upper)	
T0	0.905	0.488	0.977
T1	0.802	0.319	0.946
T2	0.815	0.359	0.949

^1^ Intraclass correlation coefficient.

## Data Availability

Anonymous raw data can be provided upon request.

## Abbreviations

The following abbreviations are used in this manuscript:

Natural killer cells	NK cells
Tumor microenvironment	TME
Myeloid-derived suppressor cells	MDSCs
Interleukin-6	IL-6
Vascular endothelial growth factor	VEGF
Matrix metalloprotease	MMP
Physical Activity Readiness Questionnaire	PAR-Q
International Physical Activity Questionnaire	IPAQ
Cardio Pulmonary Exercise Testing	CEPT
Peak aerobic capacity	VO2peak
Rate of perceived exertion	RPE
Phosphate-buffered saline	PBS
TRIS-buffered saline	TBS
Bovine albumin	BSA
3,3′Diaminobenzidine	DAB
Chemokine C-X-C motif ligand 9	CXCL9
CC-chemokine ligand	15 CCL15
Intraclass correlation coefficient	ICC
Inhibitor	IH
C-X-C motif chemokine 10	CXCL10
C-X-C motif chemokine 11	CXCL11

## Appendix A

**Table A1:** Participant characteristics.

Variable	Mean	SD ^5^
**Age in *years***	46.4	9.4
**Weight in *kg***	66.2	10.2
**Height in *cm***	167.9	7.3
**IPAQ-SF category ^1^**	Minimally active N = 8 Inactive N = 3	
**PAR-Q score ^2^**	0.1	0.3
**VO_2peak_ in *mL/min/kg ^3^***	31.1	5.66
**65% VO_2peak_ in *mL/min/kg***	20.2	3.68
**50% VO_2peak_ in *mL/min/kg***	15.6	2.83
**RPE_max_^4^**	19.7	0.45
**Watt_max_**	155.9	27.49
**Watt_65%_**	101.3	17.87
**Watt_50%_**	78.0	13.74

^1^ International Physical Activity Questionnaire—short form, ^2^ Physical Activity Readiness Questionnaire, ^3^ Peak oxygen consumption, ^4^ Rate of Perceived Exertion, ^5^ Standard deviation.

**Table A2:** Densitometry results.

Protein	difT0-T1	difT1-T2	difT0-T2	difRT0-T1	difRT1-T2	difRT0-T2
**Leptin**	1466.07	0.31	453.36	0.943	667,987	629,833
**PDGF-BB**	1875.27	0.66	1233.71	702,465	0.716	502,653
**CCL5**	1.21	0.66	0.80	713,666	1.044	744,822
**MCP1**	1.42	0.64	0.91	793,319	0.773	613,088
**IL-15**	0.63	0.87	0.55	1,061,224	0.893	947,585
**Angiogenin**	0.80	0.95	0.77	689,367	1.113	767,531
**BDNF**	0.78	0.74	0.57	550,514	1.014	558,105
**TNF alpha**	0.72	0.68	0.49	1,608	1.084	1.743
**MIG (CX CL9)**	0.92	0.72	0.66	0.497	1.332	0.662
**IL-3**	0.59	0.74	0.43	0.658	1.145	0.754
**MIP-1delta (CCL 15)**	0.92	0.65	0.60	0.700	1.565	1.096
**IGFBP-1**	1.26	1.05	1.33	0.721	1.070	0.771
**NAP-2**	1.27	0.49	0.62	0.996	1.096	1.092
**EGF**	1.25	1.31	1.63	0.602	0.704	0.424
**IGFBP-2**	1.58	0.80	1.26	0.689	1.027	0.708
**Eotaxin-1 (CCL11)**	0.72	1.66	1.20	0.741	1.017	0.754
**ICAM-1(CD54)**	0.99	1.50	1.48	2.523	0.853	2.152
**TIMP-1**	1.62	0.89	1.43	0.454	1.189	0.540
**EGFR**	1.43	0.71	1.02	0.761	0.948	0.721
**TIMP-2**	1.46	0.84	1.23	0.782	1.091	0.853
**ENA-78 (CXCL5)**	1.75	0.56	0.98	0.763	1.029	0.785
**IGFBP-6**	1.45	0.92	1.33	0.880	0.875	0.770
**MIP-1-beta**	1.61	0.81	1.31	0.654	0.658	0.430
**TRAILR3**	0.07	0.48	0.04	1.355	1.032	1.398
**Adiponectin**	19.78	1.04	20.50	0.854	0.997	0.852
**MIP-3-beta**	2.05	0.81	1.67	0.562	0.939	0.528
**MSP**	1.44	0.85	1.22	0.497	0.951	0.473
**uPAR**	1.35	0.70	0.95	1.016	0.615	0.625
**ANGPT2**	1.22	1.49	1.82	0.649	1.396	0.907
**OPG**	0.59	0.99	0.58	0.580	1.096	0.636
**GRO**	0.54	1.17	0.64	0.605	0.985	0.596
**gp130**	2.35	0.96	2.26	0.387	0.965	0.374
**HCC-4**	0.32	1.39	0.44	0.585	0.847	0.496
**Il-6 R**	2788.88	1.19	3321.07	0.878	0.916	0.804

The results of the densitometry analysis are presented as concentration increases and decreases between the time points. Values above one indicate a concentration increase, while values below one indicate a concentration decrease. The columns difT0-T1, difT1-T2, and difT0-T2 refer to the exercise condition; difRT0-RT1, difRT1-T2, and difRT0-T2 refer to the control condition.
